# Synthetic Aminochalcone Prevents Hyperglycemia-Induced Anxiety and Delays Pentylenetetrazole-Induced Epileptic Crisis in Adult Zebrafish

**DOI:** 10.1021/acsomega.5c01614

**Published:** 2025-07-14

**Authors:** Arnaldo Solheiro Bezerra, Amauri Barbosa da Silva Junior, Antonio Wlisses da Silva, Emanuela de Lima Rebouças Borges, Levi Magalhães Gurgel Macêdo, Yago Kayan de Souza Lima, Camila Costa de Sousa, Beatriz Helena Gama Joca, Ana Lorena Pereira Bezerra, Erick Patrick Alves Moreira, Lúcia de Fátima Mota Pernambuco, Ana Vitória Santana Garcia, Márcia Machado Marinho, Emmanuel Silva Marinho, Damião Sampaio de Sousa, Paulo Nogueira Bandeira, Francisco Ferdinando Mesquita Cajazeiras, Hélcio Silva dos Santos, Maria Izabel Florindo Guedes

**Affiliations:** † Graduate Program of Biotechnology, 67843 State University of Ceara, Fortaleza, Ceara 60714-903, Brazil; ‡ Graduate Program in Natural Sciences, 67843 State University of Ceara, Fortaleza, Ceara 60714-903, Brazil; § Chemistry Course, State University of Acaraú Valley, Sobral, Ceara 62010-295, Brazil; ∥ Department of Analytical and Physical Chemistry, Federal University of Ceara, Fortaleza, Ceara 60714-903, Brazil; # Mauricio de Nassau University Center, Fortaleza, Ceara 60714-903, Brazil; ¶ Chemical-pharmacological and Environmental Bioassay Laboratory - LABQFAM, Fortaleza, Ceara 60714-903, Brazil; ∞ Laboratory of Biotechnology and Molecular Biology, 67843 State University of Ceara, Fortaleza, Ceara 60714-903, Brazil

## Abstract

Neurological disorders are a major contributor to disability-adjusted life years (DALYs) worldwide, with anxiety and epilepsy being particularly prevalent. Among them, anxiety is among the top ten diseases and causes an annual cost of 42.3 billion dollars in the United States. It can also increase the chance of developing diabetes, regardless of cardiometabolic and sociodemographic factors. Diabetes, if left uncontrolled for long periods, can lead to serious health problems and, in 2021, was responsible for the deaths of 1.6 million people and affected around 10.5% of the world population. chalcones belong to the class of flavonoid compounds and have gained prominence in recent years for presenting a wide range of physiological effects, with similar effects also being found in the literature when these compounds are synthesized. Therefore, the work aimed at the synthesis, characterization, toxicological, anxiolytic, hypoglycemic, and anticonvulsant analysis of the compound 4AAF in **Danio rerio*.* Initially, amino chalcone was synthesized employing the Claisen–Schmidt condensation reaction, and then its toxicity was tested over 96 h at concentrations of 0.1, 0.5, and 1.0 mg/mL. Subsequently, groups of 6 treated animals were formed to evaluate their effects in the following tests: light and dark, novel tank test, open field test, spinning task, anxiolytic action mechanism via GABA and serotonergic, chronic hyperglycemia, reactive oxygen species, and pentylenetetrazol-induced seizure. The compound produced was shown to be nontoxic, and anxiety tests were promising at concentrations of 0.5 mg/mL, showing that aminochalcone acts on both the GABA and serotonergic pathways and that it causes a reduction in the locomotor pattern of the animals. In the context of hyperglycemia, the concentration of 0.5 mg/mL significantly reduced blood glucose levels and oxidative stress in the liver, in addition to causing anxiolytic and sedative behavior. In the seizure test, the 1.0 mg/mL concentration partially reversed PTZ-induced convulsive behavior in stage three. Finally, synthetic chalcones have more than one physiological effect, requiring bioprospecting for other possible actions, and zebrafish become an interesting model to investigate their various uses.

## Graphical abstract

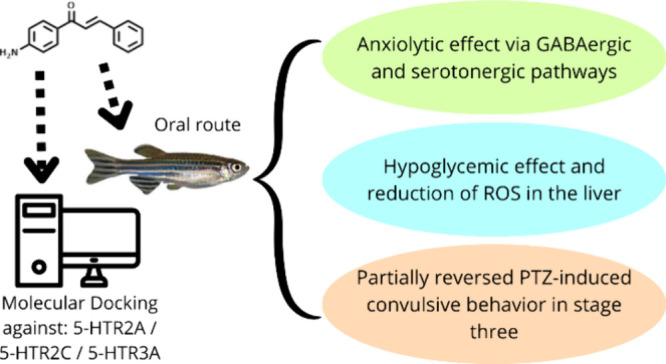

## Introduction

Neurological disorders are a major contributor to disability-adjusted life years (DALYs) worldwide, with anxiety and epilepsy being particularly prevalent. Anxiety disorders affect over 300 million people globally, while epilepsy impacts approximately 70 million individuals.^1^ Among psychiatric conditions, anxiety disorders are described as one of the ten leading causes of disability worldwide, affecting approximately 18% of the population in the United States and generating an associated annual cost of 42.3 billion dollars. In the European Union, anxiety disorders affect more than 60 million people, making them the most common psychiatric condition in the region.^2^ Brazil has the highest global prevalence of anxiety disorders and is the fifth country with the highest rates of depression.^3^

Anxiety is linked to alterations in various neurotransmission pathways, including the noradrenergic, dopaminergic, nitrergic, serotonergic, and GABAergic systems.^4,5^ Standard treatments include benzodiazepines, which have an affinity for the GABA receptor and selectively inhibit serotonin reuptake (SSRIs).^6–8^ However, long-term benzodiazepine use leads to tolerance, and abrupt discontinuation can trigger withdrawal syndrome.^9^ Similarly, chronic SSRI use is associated with significant side effects,^10^ such as amnesia and central respiratory depression.^7^ Given these challenges, there is a pressing need for novel anxiolytic and antidepressant compounds with reduced adverse effects.^11^

The GABAergic system acts in the pathophysiology and treatment of anxiety and epilepsy. GABA agonists have anticonvulsant effects, while GABA antagonists can induce seizures.^12^ Diazepam, a widely used treatment, acts nonselectively on four GABA receptor subunits, producing antiepileptic, sedative, and muscle relaxant effects.^13^ Furthermore, serotonin, an essential neurotransmitter in the central nervous system (CNS), regulates several cognitive and noncognitive functions, which include emotional responses, sleep, mood, memory, appetite, and anxiety.^14^

Anxiety is also associated with an increased risk of developing diabetes, a relationship influenced by factors such as obesity, cardiometabolic disorders, an unhealthy lifestyle, and sleep disturbances. Studies indicate that anxiety increases the risk of diabetes by 1.47 times, independent of cardiometabolic and sociodemographic factors,^15^ while a systematic review found a 19% higher risk.^16^

Diabetes is a chronic disease that causes insufficient insulin production, leading to hyperglycemia. If left unchecked, it can lead to serious complications, including blindness, kidney failure, heart attacks, strokes, and limb amputadtions. In 2021, it was responsible for the deaths of 1.6 million people,^17^ and currently affects around 10.5% of the world’s population.^18^ The link between anxiety and diabetes is complex, potentially arising from a shared genetic basis^19^ or physiological mechanisms influenced by anxiety. Additionally, the emotional burden of a diabetes diagnosis and the weight of daily tasks related to care can also increase the level of anxiety of diabetic patients,^20^ generating a possible impact on the assessment of personal health and poor glycemic control or nonadherence to medication.^21^

Chalcones, a subclass of flavonoids with a 1,3-diaryl-2-propen-1-one backbone, are secondary metabolites found in plant pigments, including those in petals, heartwood, leaves, fruits, and roots.^22^ They can also be synthetically produced via the Claisen–Schmidt condensation reaction, a widely used method due to its cost-effectiveness and simplicity.^23^ The biological activity of chalcones can be altered depending on the combination of their two aromatic rings, allowing the development of molecules with diverse pharmacological properties.^24^ Synthetic chalcones have demonstrated anxiolytic,^25–27^ antioxidant, anti-inflammatory, neuroprotective,^6^ antinociceptive, and hypoglycemic^28^ effects.

Zebrafish (*Danio rerio*) have proven to be a crucial preclinical model in drug discovery. It has high genetic similarity to the human species, with 70% of the genome preserved and more than 80% similarity to proteins related to human diseases.^29^ Their shared pharmacology with humans makes them practical for studying neurological and metabolic disorders.^30^ The zebrafish model’s advantages include its reduced size, transparency, low cost, rapid development cycle, highly conserved genes, and ease of genetic modification.^31,32^ In the neurological field, it presents behavioral patterns, a basic structure similar to the human brain, a blood–brain barrier, and neurotransmitters for identifying the pathways involved in regulating anxiety and drugs for screening before testing in rodents^31,33,34^ for the GABA and serotonergic pathways.^35,36^ Zebrafish have also been used to study metabolic diseases such as obesity and diabetes,^37^ with previous studies investigating the effects of chronic hyperglycemia induced by glucose and sucrose in adult fish.^38,39^ Zebrafish absorb glucose through glucose transporters (GLUT) in the gills and intestines.^40^

In parallel, computational studies have become essential tools for drug discovery. Molecular docking is a widely used technique that helps to elucidate the pharmacological action of therapeutic molecules for the CNS.^41^ This method effectively predicts binding modalities and calculates binding free energy between target proteins and potential drug candidates.^42^ Recent advances have identified new chalcones with anxiolytic, antinociceptive, and anticonvulsant properties.^28,43,44^

The model has proven effective in tests for both natural products^35,45,46^ and synthetic chalcones.^26–28^ However, this study aimed to perform the synthesis, characterization of the synthetic chalcone (*E*)-1-(4-aminophenyl)-3-phenylprop-2-en-1-one and evaluation of its toxicological, anxiolytic, hypoglycemic, and anticonvulsant effects in adult zebrafish.

## Results

### Acute Toxicity

After 96 h of analysis, all animals exposed to concentrations of 0.1, 0.5, and 1.0 mg/mL of aminochalcone 4AAF and the vehicle (DMSO 3%) survived, without presenting apparent anatomical changes, indicating that 4AAF was not toxic to adult zebrafish (LC50 > 1.0 mg/mL) during this period of analysis.

### Anxiolytic and Sedative Activity

#### Novel Tank Test

The new tank test was employed to evaluate the anxiety-like behavior of zebrafish after treatment with aminochalcone 4AAF. The one-way ANOVA statistical test showed significance for the total distance traveled parameter (# *p* < 0.05 vs 4AAF 0.1 mg/mL) (Figure 1 B), distance traveled in the upper portion (*p < 0.05 vs control) (Figure 1 D), latency to enter the upper portion (*p < 0.05 vs control, Figure 1 E), and total time in the upper portion of the aquarium (*p < 0.05 vs control, Figure 1 F). Dunn’s multiple comparison test showed that fish treated with the highest concentrations of 4AAF exhibited anxiolytic behavior, exploring the upper part of the tank longer and more intensely (*p < 0.05 vs control), as well as having an increased latency to enter the upper part of the tank (*p < 0.05 vs control), an indication of sedation.

**Figure 1:**
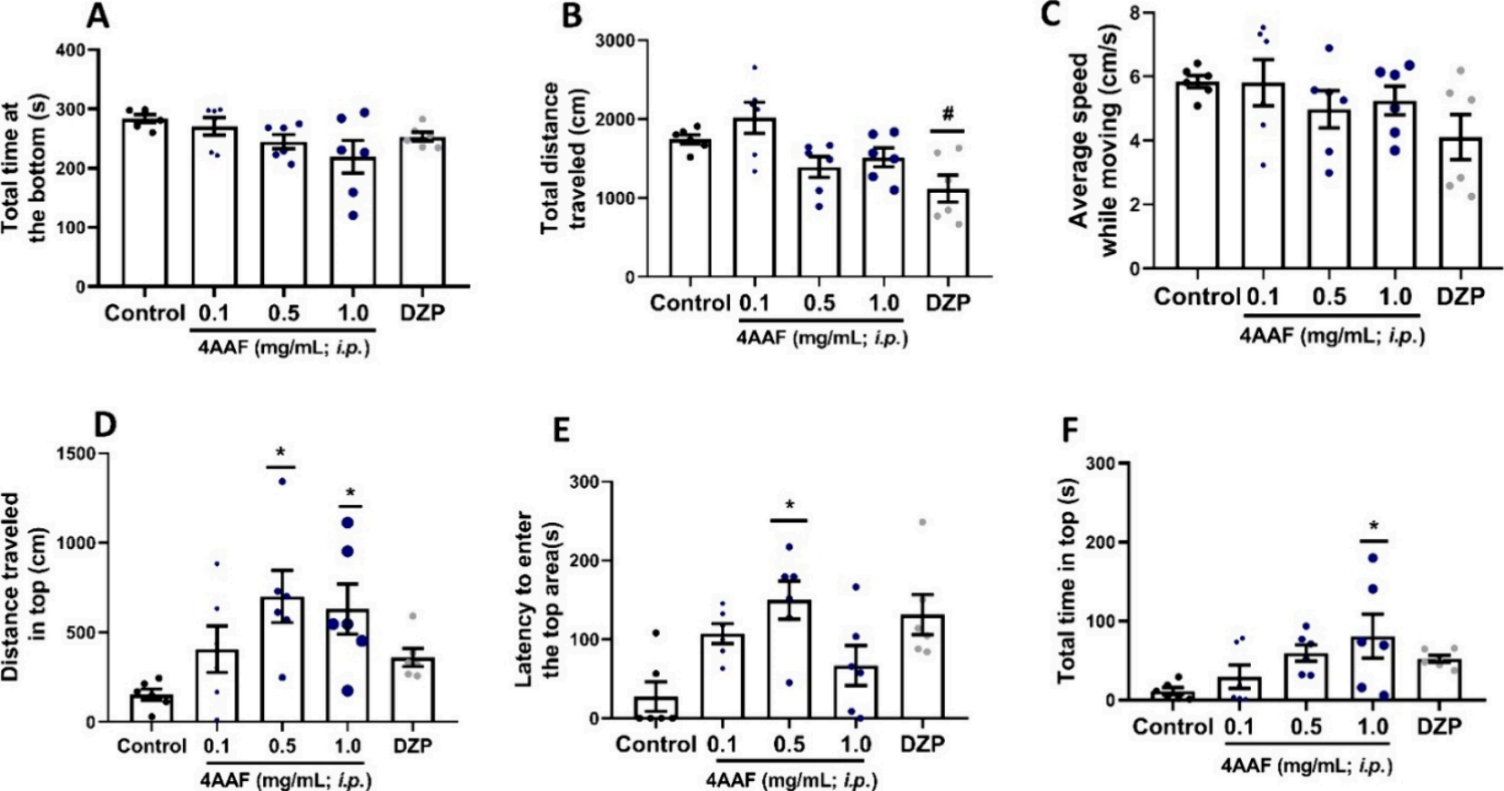
Effect of amino chalcone 4AAF on the evaluation of locomotor activity and anxiety behavior in adult zebrafish using the Novel Tank test for 5 min (*n* = 6 fish/group). (A) Distance traveled on the bottom (cm), (B) total distance traveled (cm), (C) average speed in movement (cm/s), (D) distance traveled on the top (cm), (E) latency to enter the top area (s), and (F) total time on the top (s). DZP—diazepam (0.1 mg/mL, p.o.); Control—DMSO 3%. Data are expressed as mean ± SD (graphs A–D), and as median and I.Q.R. (graphs E and F). ANOVA for B and D, Kruskal–Wallis for E and F (* *p* < 0.05 vs Control; # *p* < 0.05 vs 4AAF 0.1 mg/mL).

### Open Field Test and Spinning Task

The 0.5 mg/mL concentration of 4AAF and diazepam (* *p* < 0.05; *** *p* < 0.001 vs control) caused a significant impairment of motor function in adult zebrafish compared to the control group in the open field test, reducing the number of line crossings in the Petri dish (Figure 2A). In addition, the 0.1 mg/mL concentration of 4-AAF and diazepam (** *p* < 0.01; **** *p* < 0.0001 vs control) decreased the latency to enter the vortex in the rotation task compared to the control group (Figure 2B). These results suggest a sedative effect of 4AAF similar to that of diazepam.

**Figure 2:**
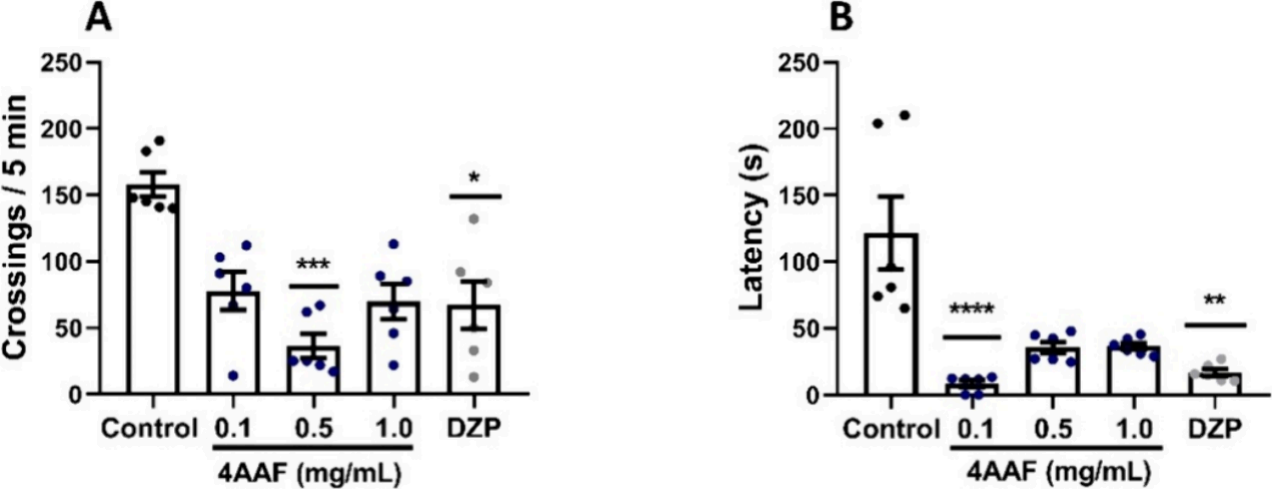
Effect of amino chalcone 4AAF on the evaluation of zebrafish locomotor activity in the open field test for 5 min (A) and in the spinning task (B) (*n* = 6 fish/group). DZP—diazepam (0.1 mg/mL, p.o.); Control—DMSO 3%; Naive—animals without treatment. The data are expressed as median and I.Q.R. (graphs A and B). Kruskal–Wallis for A and B (* *p* < 0.05; ** *p* < 0.01; *** *p* < 0.001; **** *p* < 0.0001 vs Control).

### Light and Dark

Aminochalcone at all concentrations tested and diazepam significantly reduced anxiety-like behavior (*p < 0.05, **p < 0.01, ***p < 0.001, ****p < 0.0001 vs Control) in adult zebrafish. This effect can be observed when comparing the time spent in the illuminated region of the aquarium, which in the reported groups increased significantly compared to animals in the negative control group (Figure 3A). In addition, the highest concentrations of 4AAF evaluated altered the latency time (**p < 0.01, ***p < 0.001, ****p < 0.0001 vs Control; Figure 3B) and the number of crossings between the light and dark sides (*p* > 0.05 vs Control; Figure 3C) when compared to animals in the control group, demonstrating that there were changes in the locomotor pattern and exploratory capacity of the fish, similar to the group treated with the drug diazepam. This confirms the sedative effects and motor impairment observed in previous analyses.

**Figure 3:**
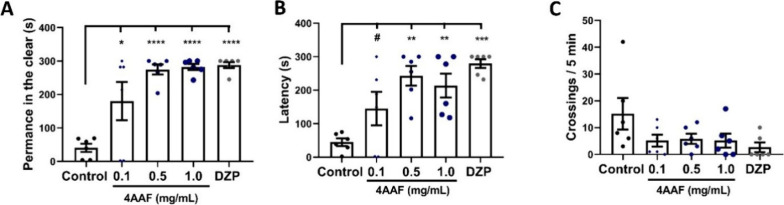
Effect of aminochalcone 4AAF on anxiety-like behavior (A), latency to enter the dark area of the aquarium (B), and line crossings (C) in the light and dark test. DZP—diazepam (0.1 mg/mL, p.o.); Control—DMSO 3%. The values presented correspond to the mean ± standard error of the mean for six animals/group, using a one-way ANOVA test followed by Tukey’s test (* *p* < 0.05, ** *p* < 0.01, *** *p* < 0.001, **** *p* < 0.0001 vs Control; # *p* < 0.05 vs DZP).

### Evaluation of the Mechanism of Action (GABA_A_ System)

The interaction between 4AAF and the GABAA receptor was evaluated by pretreatment with flumazenil (a benzodiazepine inhibitor of the GABAA receptor). As a result, one-way ANOVA statistical analysis indicated that flumazenil acted by inhibiting (^####^ *p* < 0.0001 vs 4AAF) the anxiolytic and sedative behavior of 4AAF (0.5 mg/mL), causing a reduction in the time spent in the illuminated area of the aquarium and latency, similar to what occurred with the group treated with DZP (#### *p* < 0.0001 vs DZP) (Figure 4A,B). Furthermore, pretreatment with flumazenil restored the locomotor pattern in the group treated with 4AAF and DZP (*p* > 0.05) (Figure 4C).

**Figure 4:**
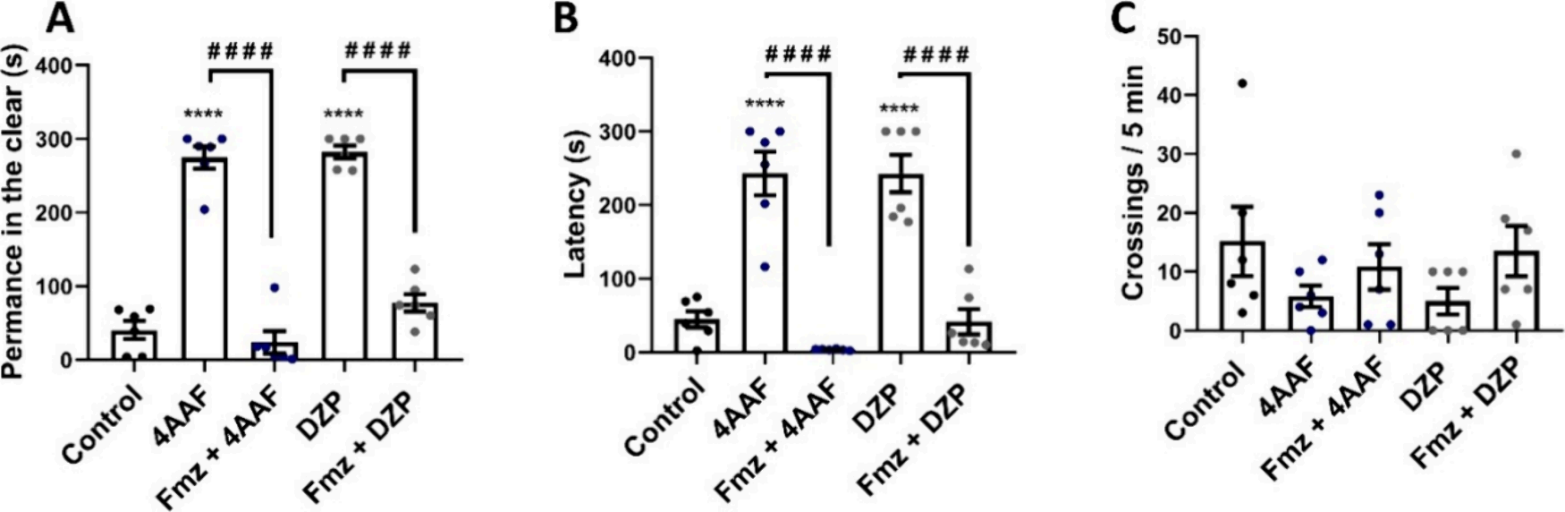
Effect of pretreatment with Fmz—flumazenil (0.1 mg/mL) on the anxiolytic behavior of animals treated with aminochalcone 4AAF (0.5 mg/mL). Light-remaining (A), latency to enter the dark area of the aquarium (B), and line crossings (C) in the light and dark test. DZP—diazepam (0.1 mg/mL, p.o.); Control—DMSO 3%. The values presented correspond to the mean ± standard error of the mean for six animals/group, using a one-way ANOVA test followed by Tukey’s test (**** *p* < 0.0001 vs Control; ^####^ *p* < 0.0001 vs DZP or chalcone).

### Evaluation of the Mechanism of Action (Serotonergic System)

The interaction with the serotonergic pathway was analyzed by pretreatment with cyproheptadine (5-HTR_2A_ antagonist), pizotifen (5-HTR_1/2*A*/2C_ antagonist), and granisetron (5-HTR_3A/3B_ antagonist). Cyproheptadine, pizotifen, and granisetron reversed (^####^ *p* < 0.0001 vs 4AAF) the anxiolytic behavior of animals treated with 4AAF (0.5 mg/mL) and fluoxetine (^####^ *p* < 0.0001 vs Fluoxetine) by significantly reducing the time the fish spent in the light area of the aquarium (Figure 5).

**Figure 5:**
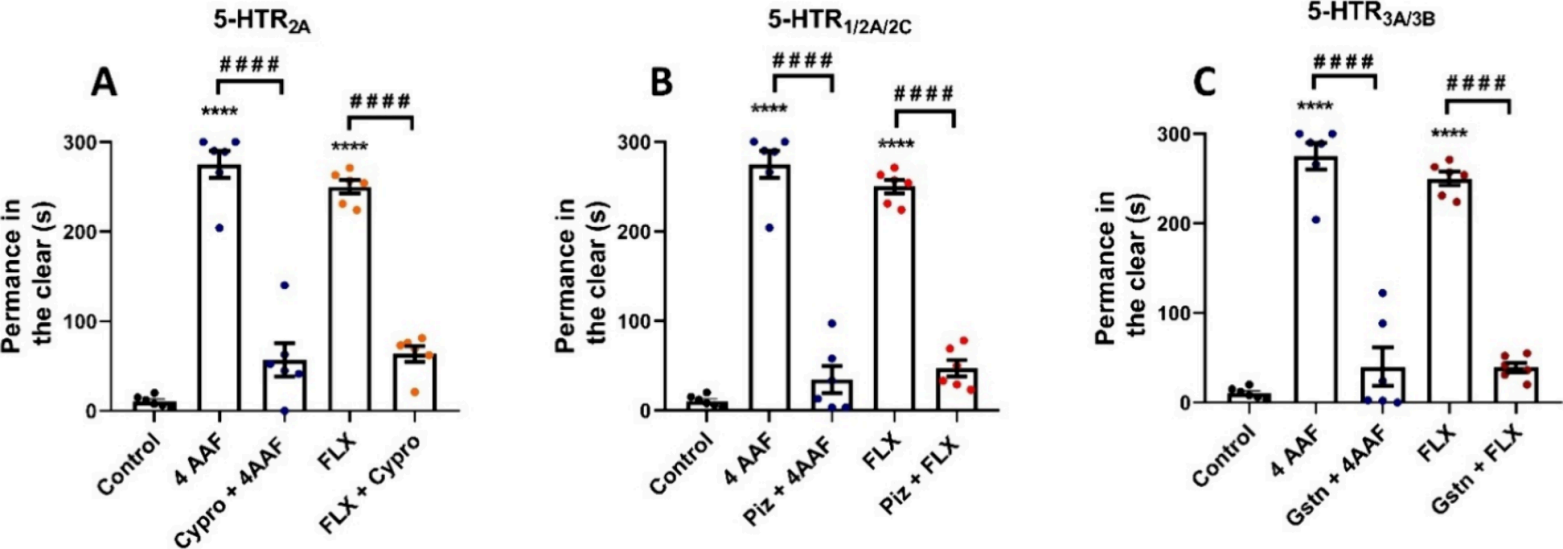
Effect of serotonergic antagonists on the anxiolytic behavior of animals treated with chalcone 4AAF (0.5 mg/mL). Remaining in the light in the light and dark test. FLX—fluoxetine; Control—DMSO 3%. The values presented correspond to the mean ± standard error of the mean for six animals/group, using a one-way ANOVA test with antagonists followed by Tukey’s test (****p < 0.0001).

### Molecular Docking

#### Molecular Docking Against 5-HTR_3A_

Given the independent simulations, it can be established that the proposed ligands fit the parameters with RMSD lower than 2.0 Å^2^ (Table 1), indicating that the widespread simulation protocol promotes ligand selectivity for the receptor, with emphasis on the chalcone 4AAF, which presents a value (RMSD = 1.359) higher than the antagonist CWB (granisetron) with RMSD = 1.664.

**Table 1:** Data on Ligand–Receptor (L–R) Interactions in the Redocking Process of the Antagonist CWB and Molecular Docking Simulations of the 4AAF Chalcone, via 5-HTR_3A_ ^a^

compd	RMSD	Δ*G*	residue	dis_(L–R)_ (Å)	inter. type
CWB^b^	1.664	8.1	Ile44E	3.79	hydrophobic
			Trp63E	3.71	hydrophobic
			Arg65E	3.76	hydrophobic
			Trp156A	3.65	hydrophobic
			Phe199A	3.53	hydrophobic
			Tyr207A	3.56	hydrophobic
			Arg65E	4.12	π-cation
			Arg65E	4.40	π-cation
4AAF	1.359	–7.9	Ile44E	3.93	hydrophobic
			Trp63E	3.65	hydrophobic
			Trp63E	3.93	hydrophobic
			Arg65E	3.60	hydrophobic
			Tyr126E	3.92	hydrophobic
			Pro128E	3.92	hydrophobic
			Trp156A	1.72	hydrophobic
			Trp156A	0.97	hydrophobic
			Trp63E	3.82	π-stacking
			Trp63E	3.88	π-stacking
			Trp156A	3.61	π-stacking
			Arg65E	4.11	π-cátion

a These include: RMSD statistical adjustment, affinity energy (Δ*G*), interaction amino acid residues, ligand–receptor distance (dis­(L–R)), and types of interaction.

b The agonist used in molecular docking simulations.

Although chalcone 4AAF has a higher RMSD value, it demonstrates a small variation in the affinity energy (Δ*G* = −7.9 kcal/mol), while CWB exhibits a higher energy with a value of −8.1 kcal/mol, thus both ligands exhibited values of Δ*G* < −6.0 kcal/mol (Table 1), that is, within the favorable energy criteria.

Considering the nature of the interactions of the ligands in question, it was possible to infer that the chalcone 4AAF presents an analog to the catalytic occupation site of the setron CWB (granisetron), characterized by the interaction of the amino acid residues found in the 5-HTR_3A_ subunits (Figure 6A). This observation raises the possibility of modulation of the 5-HTR_3A_ in line with the action of other setrons, highlighting the hydrophobic interactions shared by the compounds (CWB and 4AAF) with the residues Ie44E, Trp63E, π-cation Trp63E, and π-stacking with Trp156 (Figure 6B). The similarity in question is discernible in the heatmap presented in Figure 6C, where it is highlighted that the ligands establish moderate intensity interactions with the residues Ile44E, Trp63E, and Arg65E, with distances calculated around 3.5 Å, while Trp156A showed a more pronounced interaction (dis­(L–R) > 0.5 Å).

**Figure 6:**
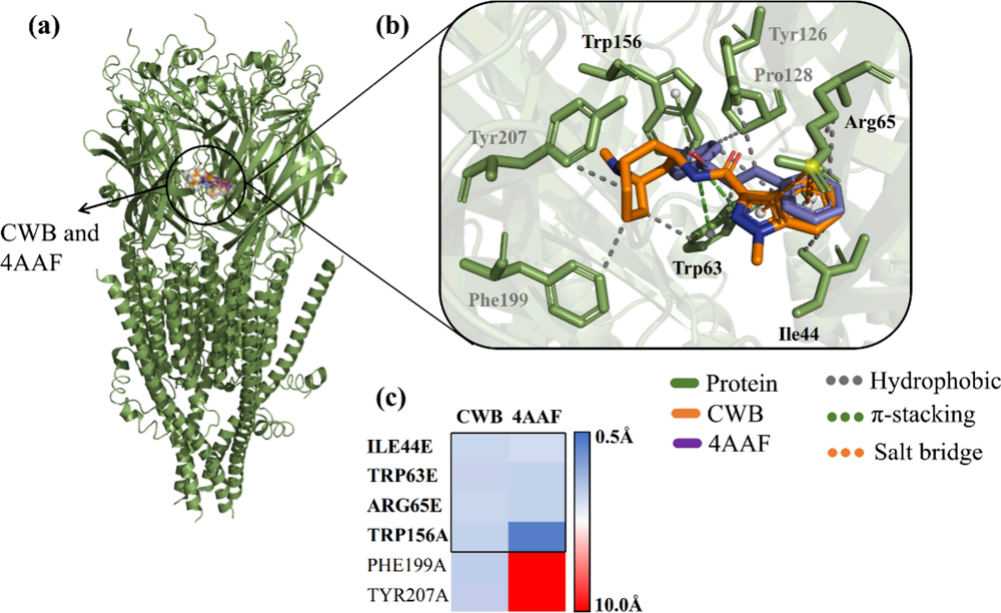
(A) Three-dimensional map of 5-HTR_3A_ with CWB (orange) and 4AAF (purple), (B) visualization of the binding site of setrons and 4AAF exposing the types of bonds, and (C) heat map of the interaction between the ligand and the receptor from the calculated distance.

### Molecular Docking Against 5-HTR_2C_

The mechanism employed for the simulations of the ligands to the 5-HTR_2C_ corroborates the values of Δ*G* < −6.0 kcal/mol and RMSD below 2.0 Å^2^ (reference values) (Table 2). Likewise, it establishes that chalcone 4AAF provides selectivity to the 5-HTR_2C_ receptor with an emphasis on the RMSD value (1.143) higher than that of the E2J agonist (ritanserin), which obtained an RMSD of 1.994. Although chalcone 4AAF demonstrates a slightly higher RMSD value, it is pertinent to note that its variation in affinity energy (Δ*G* = −7.7 kcal/mol) is significant, while E2J presents a higher energy, with a value of −9.8 kcal/mol (Table 2).

**Table 2:** Data on Ligand–Receptor (L–R) Interactions in the Redocking Process of the Agonist E2J and Molecular Docking Simulations of the 4AAF Chalcone, via 5-HTR_2C_ ^a^

compd	RMSD	Δ*G*	residue	dis_(L–R)_(Å)	inter. type
E2J^b^	1.994	–9.8	Trp130A	3.69	hydrophobic
			Ile142A	3.75	hydrophobic
			Ala222A	3.87	hydrophobic
			Phe223A	3.75	hydrophobic
			Phe328A	3.34	hydrophobic
			Tyr358A	3.92	hydrophobic
			Trp324A	4.89	π-stacking
			Phe214A	3.74	halogen bond
			Asp134A	3.07	salt bridge
4AAF	1.143	–7.7	Val135A	3.98	hydrophobic
			Ile142A	3.78	hydrophobic
			Leu209A	3.77	hydrophobic
			Val215A	3.65	hydrophobic
			Ala222A	3.97	hydrophobic
			Phe223A	3.81	hydrophobic
			Leu209A	3.82	hydrophobic
			Ser138A	2.69	H-bond
			Thr139A	3.33	H-bond
			Trp324A	5.24	π-stacking
			Phe328A	5.01	π-stacking

a These include: RMSD statistical adjustment, affinity energy (Δ*G*), interaction amino acid residues, ligand–receptor distance (dis­(L–R)), and types of interaction.

b The agonist used in molecular docking simulations.

Analysis of ligand interactions revealed that the 4AAF chalcone occupies a position similar to that of the catalytic site of E2J (ritanserin) and interacts with amino acid residues located in the 5-HTR_2C_ subunit (Figure 7A). These findings raise the intriguing possibility that the 5-HTR_2C_ receptor may be modulated by other chalcone analogues, highlighting the hydrophobic nature of the binding of E2J and 4AAF to residues Ile42A, Ala222A, and Phe223A (Figure 7B). A striking similarity can be observed in the heatmap shown in Figure 7c, where it is highlighted that the ligand interacts with these residues with moderate intensity while maintaining a calculated distance of approximately 3.5 Å.

**Figure 7:**
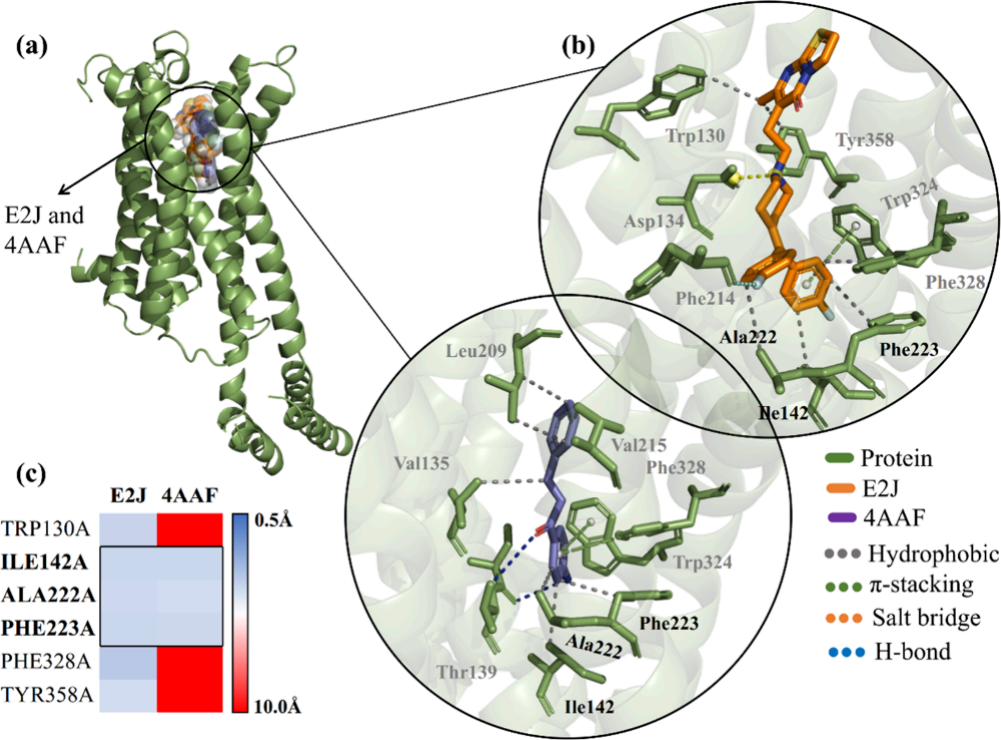
(A) Three-dimensional map of the 5-HTR_2C_ with E2J (orange) and 4AAF (purple), (B) visualization of the binding site of the E2J and 4AAF exposing the types of bonds, and (C) heat map of the interaction between the ligand and the receptor from the calculated distance.

### Molecular Docking Against 5-HTR_2A_

The ligands used in the 5-HTR_2A_ simulations demonstrated that chalcone 4AAF has a higher RMSD value (0.182) than that of the agonist 8NU (risperidone), which obtained an RMSD of 1.628 (Table 3). However, the variation in the affinity energy of risperidone is high with a value of −11.1 when compared to chalcone 4AAF with −8.2, respectively, that is, values of Δ*G* < −6.0 kcal/mol and RMSD below 2.0 Å^2^ (reference values) (Table 3).

**Table 3:** Data on Ligand–Receptor (L–R) Interactions in the Redocking Process of the Agonist E2J and Molecular Docking Simulations of the 4AAF Chalcone, via 5-HTR_2A_ ^a^

compd	RMSD	Δ*G*	residue	dis_(L–R)_ (Å)	inter. type
8NU^b^	1.628	–11.1	Trp151A	3.47	hydrophobic
			Phe332A	3.75	hydrophobic
			Trp336A	3.54	hydrophobic
			Phe339A	3.89	hydrophobic
			Leu362A	3.58	hydrophobic
			Trp336A	4.64	π-stacking
			Trp336A	5.04	π-stacking
			Phe340A	4.75	π-stacking
			Asp155A	3.09	salt bridge
4AAF	0.182	–8.2	Val156A	3.71	hydrophobic
			Val156A	3.63	hydrophobic
			Pro209A	3.62	hydrophobic
			Ile210A	3.58	hydrophobic
			Ile210E	3.89	hydrophobic
			Phe234A	3.61	hydrophobic
			Phe234A	3.79	hydrophobic
			Ser159A	2.24	H-bond

a These include RMSD statistical adjustment, affinity energy (Δ*G*), interaction amino acid residues, ligand–receptor distance (dis­(L–R)), and types of interaction.

b The agonist used in molecular docking simulations.

Although the protein (5-HTR_2A_)–ligands (8NU and 4AAF) complex shows similar energetic and statistical patterns, the interactions of chalcone 4AAF were not significant, given the absence of interactions with the catalytic domain, presenting hydrogen bonding with Ser159, hydrophobic interactions with Val156, Ile210, Pro209, and Phe234 (Figure 8C). Meanwhile, risperidone (8NU), the reference agonist for molecular docking simulations, shows hydrophobic interactions with Trp151, Leu352, Phe339, and Phe332, π-stacking with Phe340 and Trp336, and a salt bridge with Asp155 (Figure 8B). It is worth noting that chalcone 4AAF, due to its conformation to the complex, can act as a positive allosteric modulator, increasing its receptor activity.

**Figure 8:**
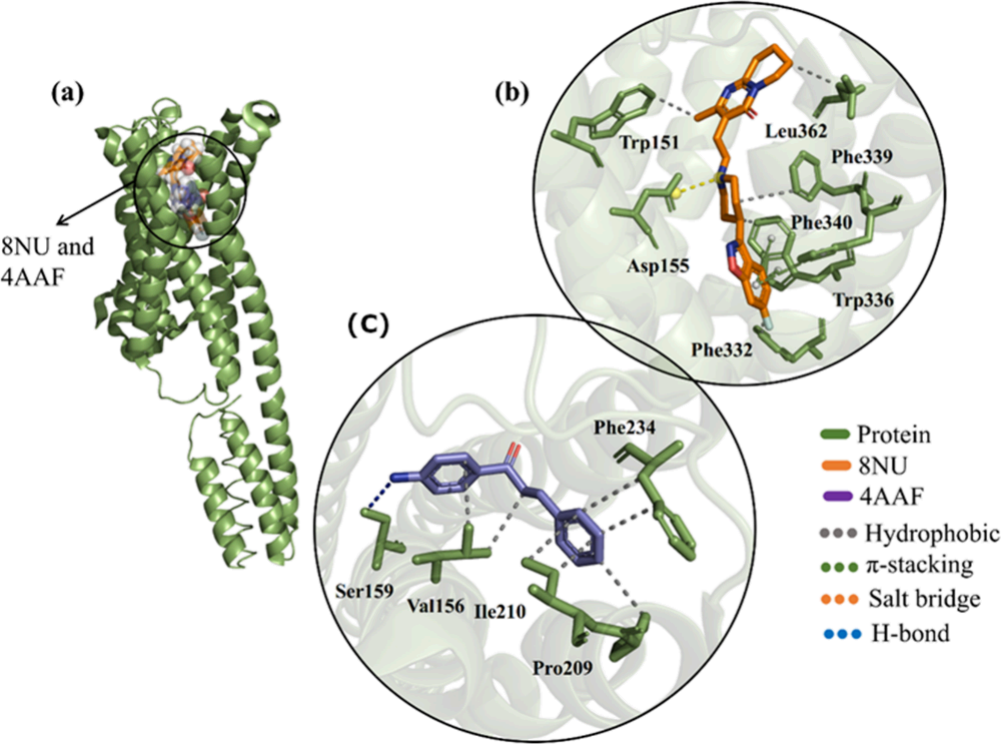
(A) Three-dimensional map of the 5-HTR_2A_ with 8NU (orange) and 4AAF (purple), (B) visualization of the binding site of the 8NUand 4AAF exposing the types of bonds, and (C) heat map of the interaction between the ligand and the receptor from the calculated distance.

### 3.4 Hyperglycemia, Anxiety, and ROS Analysis

The effect of aminochalcone on sucrose-induced anxiety and hyperglycemia was investigated. In the light and dark test, hyperglycemic fish (control group) remained in the dark for most of the time (Figure 9A), had reduced latency time to enter the dark (Figure 9B) and a greater number of crossings from the light to the dark side (Figure 9C), unlike the group treated with 4AAF that spent most of the analysis time in the light area of the aquarium (**p < 0.01 vs control), increased latency time and reduced the number of crossings from the light to the dark. Metformin had no anxiolytic or sedative effects. In addition, aminochalcone and metformin significantly reduced sucrose-induced hyperglycemia (*p < 0.05; **p < 0.01 vs control, Figure 9D), with mean blood glucose levels of 92.6 ± 48.7 in the negative control group (3% DMSO), 27.6 ± 9.6 mg/dL in the 4AAF-treated group and 26.6 ± 9.6 mg/dL in the metformin-treated group. 4AAF also prevented (***p < 0.001 vs control) hyperglycemia-induced oxidative stress in liver and brain tissues of adult zebrafish (Figure 9E,F).

**Figure 9:**
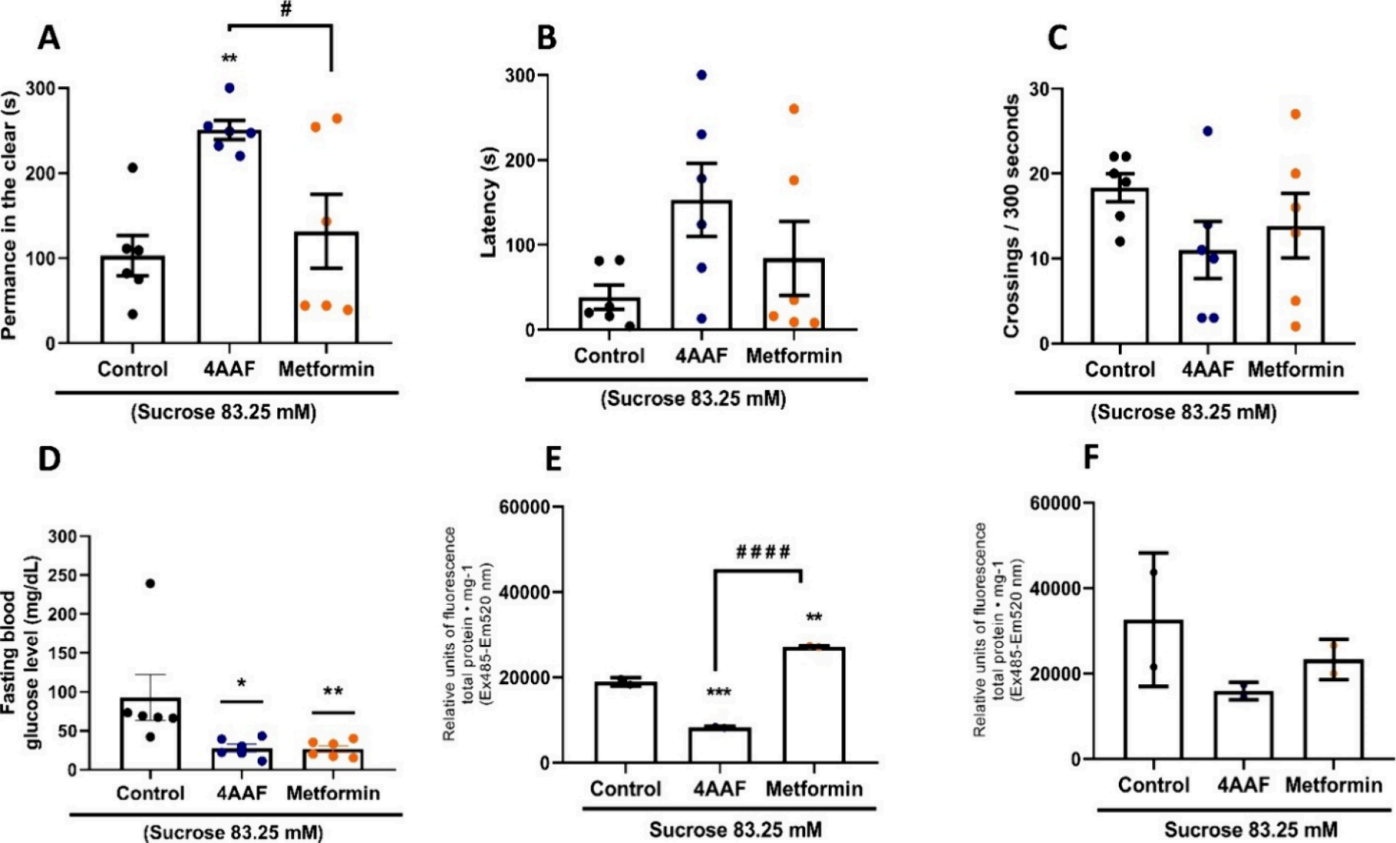
Effect of chalcone 4AAF (0.5 mg/mL) on anxiety induced by subchronic hyperglycemia (7 days) induced by sucrose. Light-time (A), latency to enter the dark area of the aquarium (B), and line crossings (C) in the light and dark test. Glycemic level (D), ROS of liver tissues (E), and ROS of brain tissues of animals treated for 4 days after sucrose withdrawal (F) (*n* = 3 in duplicate for ROS tests). Control—DMSO 3%. Data are expressed as mean ± SD (graphs A, B, C, E, and F), and as median and I.Q.R. (graph D) (* *p* < 0.05, ** *p* < 0.01, *** *p* < 0.001, **** *p* < 0.0001 vs Control; # *p* < 0.05; # # # # *p* < 0.0001 vs metformin).

### Pentylenetetrazol (PTZ)-Induced Seizures

The highest concentration of 4AAF (1.0 mg/mL) partially reversed PTZ-induced convulsive behavior in stage three (*p* > 0.05 vs control), demonstrating an effect analogous to that of DZP (*p* > 0.05 vs control), which also delayed the clonic phase of the seizure. No effect of 4AAF or DZP was observed in stages I and II (Figure 10).

**Figure 10:**
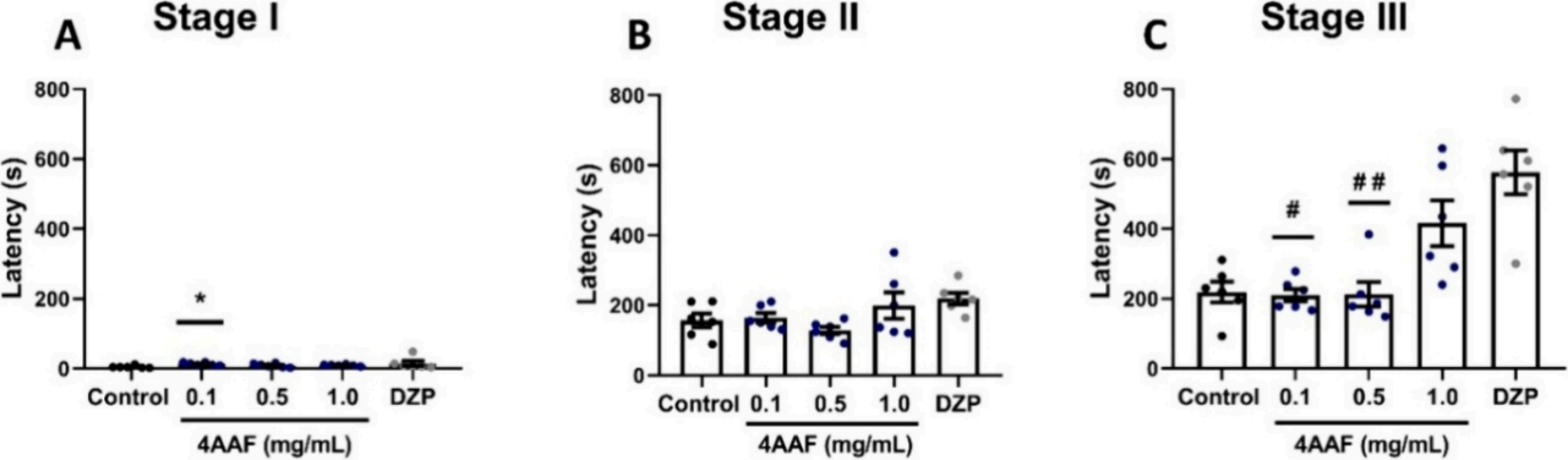
Effect of aminochalcone 4AAF (0.1, 0.5, and 1.0 mg/mL) on pentylenetetrazol-induced seizures. First stage latency (A), second stage latency (B), and third stage latency (C). DZP—diazepam (0.1 mg/mL, p.o.); Control—DMSO 3%. Data are expressed as mean ± SD (graphs A and C), and as median and I.Q.R. (graph B) (* *p* < 0.05 vs Control; # *p* < 0.05, # # *p* < 0.01 vs diazepam).

## Discussion

According to the literature, this is an unprecedented study demonstrating the anxiolytic, sedative, hypoglycemic, and anticonvulsant effects of synthetic aminochalcone.

The toxicity result is in agreement with that observed in other studies,^27,28,36^ since both 4AAF and other chalcones are nontoxic at concentrations up to 40 mg/mL for 96 h of analysis.

The neurobehavioral effects of chalcones are reported, confirming the anxiolytic effect of these flavonoids.^26,43,44,47^ The effects of anxiolytic drugs typically act by increasing exploratory activity in the open field in the novel tank test,^48^ inducing a sedative effect or reducing their locomotor activity.^49^ Aminochalcone 4AAF increased the zebrafish’s exploration time and latency to enter the top of the aquarium in the novel tank test, indicating a sedative anxiolytic effect.

In order to confirm the sedative effect of 4AAF, the open field test in a Petri dish and a spinning task were performed. The open field test in a Petri dish is an example of a test that can be applied in the behavioral evaluation of drugs that exert a sedative effect on the CNS of zebrafish, reducing the animals’ locomotion.^26,43,50^ The spinning task can be considered a sensitive indicator of motor capacity in zebrafish, contributing to the expansion of the behavioral assessment repertoire and assessing subtle motor deficiencies induced by drug treatments.^51^ 4AAF drastically reduced fish locomotion and latency in entering the whirlpool in the spinning task, confirming the sedative effect of aminochalcone. Studies that analyzed anxiolytic drugs corroborate the results obtained in this study, as these compounds reduce the locomotor activity of adult zebrafish, confirming that sedative anxiolytic compounds decrease the locomotor activity of adult zebrafish.^26,49,52^ The acute toxicity analysis of 4AAF showed that none of the concentrations used were toxic during the 96 h.

When anxious, adult zebrafish instinctively avoid the light region. Therefore, in the screening of anxiolytic medications, the light/dark test analyzes the time spent on the dark side, quantifies the total number of crossings between the light and dark zones, and measures the latency to enter the dark area.^53^ The results showed that the animals that were administered aminochalcone spent more time in the light zone of the aquarium, increased latency, and reduced the number of crossings between the light and dark zones, confirming the anxiolytic/sedative effect observed in the new tank in the open field in a Petri dish and the spinning task. The effects obtained in this study are similar to those found in other studies in the literature that analyzed the anxiolytic effect of synthetic chalcones, such as that of Xavier et al.^54^ and Mendes et al.^36^

The nervous system’s main chemical messenger is gamma-aminobutyric acid (GABA), which inhibits the CNS through GABA_A_ and GABA_B_ receptors. The first is responsible for opening a chloride-sensitive transmembrane channel that inhibits neuronal activity.^55^ Flumazenil identifies the anxiolytic effect via the GABAergic pathway due to its antagonistic effect at the benzodiazepine binding site on GABA_A_ receptors.^56^ Pretreatment with flumazenil inhibited the anxiolytic behavior caused by 4AAF and diazepam. Thus, aminochalcone causes anxiolytic and sedative behavior by interacting with the GABA_A_ receptor in the same binding site as benzodiazepines. chalcones from different studies also generated anxiolytic behavior in adult zebrafish via GABA_A_.^36,43,57^

Another pathway that is involved in anxiety is the serotonergic pathway,^79^ and when the animal presents an anxiogenic pattern in the light–dark test, it is related to the presence of high extracellular levels of 5-HT in the zebrafish brain,^73^ on the other hand, with reduced levels, they generally cause anxiolytic behaviors.^80^ The 5HT1A pathway is associated with antidepressant and/or anxiolytic effects, being an inhibitory receptor coupled to the G protein. Studies that used agonists of this receptor obtained results of anxiolytic behaviors in zebrafish.^49,73,79^ The 5HTR2C receptor is correlated with eating and mood disorders, anxiety, and motor behavior,^81^ and the genetic similarity between the mammalian receptor and that of zebrafish allows the production and formulation of new drugs that act on this receptor.^82^ Furthermore, the 5-HTR3 is related to the neural processes that govern emotion and cognition, and antagonists of these receptors have been shown to be beneficial in the treatment of several psychiatric disorders,^83^ for example, depression, anxiety, schizophrenia, and drug abuse.^84^ The anxiolytic mechanism of action of amino chalcone for the serotonergic pathway, occurring through serotonin receptor antagonists prior to treatment, was also investigated—5-HTR1 and 5-HTR2*A*/2C (pizotifen), 5-HTR2A (cyproheptadine), and 5-HTR3*A*/3B (granisetron) blocked the effect of 4AAF on the anxiolytic behavior of fish, evidencing the anxiolytic effect of 4AAF through serotonergic neuromodulation. However, this is the first amino chalcone to indicate its possible anxiolytic effect through both the GABAergic and serotonergic pathways.

The predictive molecular docking study indicated that the amino chalcone may cause the anxiolytic effect through its action via serotoninergic neuromodulation, interacting with serotonin receptors 2C and 3A, binding in the same catalytic region as the agonist ritanserin (in the 5-HT2C receptor) through hydrophobic bonds with residues Ile42A, Ala222A, and Phe223A, and also binding in the catalytic site of granisetron (in the 5-HT3A receptor) through hydrophobic bonds in residues Ie44E, Trp63E, π-cation Trp63E, and π-stacking with TRp156. In addition, amino chalcone showed energetic and statistical binding patterns similar to those of ritanserin at the 5-HT2C receptor and to those of granisetron at the 5-HT3A receptor. In the 5-HT2A receptor, molecular docking indicated that amino chalcone interacts in a different region compared to the antagonist risperidone and therefore may act as a positive allosteric modulator, increasing its activity at the receptor.

In addition to the pronounced anxiolytic effect, the literature points to the action of chalcones in different pathways to promote the hypoglycemic effect, such as inhibition of carbohydrate digestion and reabsorption of the sodium glucose transporter (SGLT-2), reduction in the generation of advanced glycation end products (AGEs) and insulin mimicry. Another reported effect of chalcones is the elimination of free radicals through the hydrogen atom transfer (HAT) mechanism, which is possible through the donation of hydrogen atoms through the delocalized π electrons that hover around their aromatic structures.^58^ Aminochalcone 4AAF reduced anxiety and sucrose-induced hyperglycemia and protected brain and liver tissues from oxidative stress, unlike metformin, which did not reverse anxiety and did not prevent tissues from oxidative stress caused by sucrose-induced hyperglycemia. This reveals the pharmacological importance of Aminochalcone in anxiety and hyperglycemia.

The research into the anticonvulsant effect of amino chalcone was carried out using PTZ, a convulsive chemotherapy drug that acts allosterically on the GABAA receptor^59^ and is used in zebrafish to study epileptic-like effects.^60,61^ The result indicated the anticonvulsant effect of the highest concentration of 4AAF. Other chalcones with anxiolytic effect through GABAergic neuromodulation also demonstrated an anticonvulsant effect in adult zebrafish,^36,43^ corroborating the result of aminochalcone 4AAF.

## Conclusions

The synthetic aminochalcone 4AAF induced sedative-anxiolytic behavior in adult zebrafish, and GABA_A_ and 5-HT receptors may be involved in these effects. Molecular docking simulations showed that chalcone 4AAF has high selectivity for 5-HT_3A_ and 5-HT_2C_ receptors with relevant statistical, energetic, and interaction descriptors, and stimulates allosteric behavior toward the 5-HT_2A_ receptor, where it provides enhanced modulatory activity. In addition, aminochalcone showed anxiolytic and hypoglycemic effects, reversing sucrose-induced anxiety and hyperglycemia in adult zebrafish and delaying the clonic stage of the epileptic crisis. Aminochalcone is therefore a promising flavonoid for the development of treatments for anxiety, hyperglycemia, and seizures.

## Methods

### Drugs and Reagents

The following drugs/reagents were used throughout the experiment: dimethyl sulfoxide (DMSO 3%; Dynamic), diazepam (DZP, NeoQumica), flumazenil (Fmz; Sandoz), fluoxetine (Eli Lilly, Indianapolis, IN, USA), pizotifen maleate (Central Manipulation Pharmacy, São Paulo, SP, Brazil), granisetron hydrochloride (Corepharma, Middlesex, NJ, USA), cyproheptadine (Evidence Pharmaceutical Solutions, Fortaleza, CE, Brazil), and pentylenetetrazole (PTZ, Sigma-Aldrich).

### Synthesis and Characterization of Chalcone

The partner research group donated the amino chalcone (*E*)-1-(4-aminophenyl)-3-phenylprop-2-en-1-one used in this study (Scheme 1). The production and characterization processes are detailed in the following article.^62^ Briefly, amino chalcone was produced by the Claisen–Schmidt condensation reaction under basic conditions from the products *p*-aminoacetophenone and benzaldehyde, and stirred for 48 h. Finally, the solution was vacuum filtered and washed with cold water until pH 7.0 was reached, and then, the solution was analyzed by TLC.

**Scheme 1:**
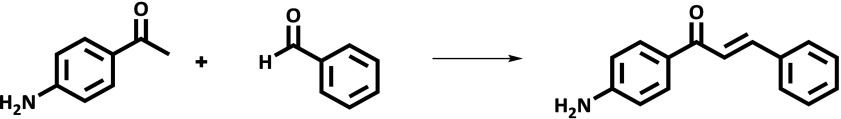
Synthesis of Aminochalcone

### Zebrafish

The zebrafish (*Danio rerio*) used in the study were obtained from the company Agroquímica: Comércio de Produtos Veterinários LTDA, a supplier in Fortaleza (Ceará, Brazil) and were wild, of both sexes, adults aged between 90 and 120 days, measuring 3.5 ± 0.5 cm in length and weighing 0.4 ± 0.1 g. Upon arrival, the animals remained in glass aquariums measuring 30 cm × 30 cm × 20 cm for 24 h in glass aquariums, where dechlorinated water (ProtecPlus) was added and maintained at a temperature of 26–28 °C, pH 7.0 and equipped with air pumps with submerged filters and a circadian cycle of 14/10 light/dark. The zebrafish were fed ad libitum up to 24 h before the experiments. After the experiments, the fish were euthanized by immersion in ice water (2–4 °C) for 1 min until the loss of opercular movements. The Ethics Committee approved all experimental procedures for using Animals of the State University of Ceará (CEUA-UECE) under protocol number 04983945/2021.

### General Protocol

Briefly, on the day of the experiments, fish were randomly selected from both sexes and transferred to a damp sponge to receive oral treatments (po), which consisted of 20 μL of the test sample concentrations (aminochalcone 4AAF). Diazepam and serotonergic antagonists were also administered orally, while fluoxetine and flumazenil (benzodiazepine antagonist) were administered intraperitoneally (i.p.). After treatment administration, fish were individually placed in beakers (250 mL) containing 150 mL of aquarium water to rest. Insulin syringes (0.5 mL; UltraFine BD) with a 30G needle were used for i.p. treatments, and an automated pipette was used for p.o. applications. Different zebrafish were selected for each new experiment. At the end of the experiment, euthanasia occurred, as mentioned in the zebrafish section.

### Acute Toxicity

In the acute toxicity test, the animals were divided into groups (*n* = 6/group), in which each aquarium was a different treatment group and remained under analysis for 96 h. The groups of fish were treated orally with 20 μL of 4-AAF (at concentrations of 0.1, 0.5, and 1.0 mg/mL in each group per animal). A control group was treated p.o. with 20 μL of 3% DMSO (diluent of the aminochalcone concentrations).^11,54^ The acute toxicity of aminochalcone 4AAF was assessed by behavioral observations and mortality counts during the 96 h analysis period to determine whether any of the 4AAF concentrations would be lethal to 50% of the animals (LC50); the protocol used follows the guidelines of the Organization for Economic Cooperation and Development (OECD).^63^ The LC50 was determined by using the Spearman–Karber method, trimmed with 95% confidence intervals based on the number of dead fish in each group.

### Novel Tank Test

Moreira protocol^64^ with adaptations was used to evaluate the swimming behavior of fish in the new test tank. The fish were placed individually in glass aquaria measuring 30 × 30 × 15 cm in height, width, and depth, respectively, filled with 9 L of dechlorinated water (ProtecPlus). All sides of the aquarium were covered with sheets of white paper to allow recording of the animals and to minimize external influences on animal behavior, except for the front wall. In the analysis program, the aquarium was virtually divided into three equal horizontal areas (bottom, middle, and top), and the distance from the video camera was 40 cm. Groups of fish (*n* = 6/group) were pretreated (20 μL; po) with 4AAF (0.1; 0.5; 1.0 mg/mL), diazepam (DZP; 1.0 mg/mL), or vehicle (control; 3% DMSO). After 60 min of oral treatment, each fish was individually filmed for 5 min to assess: (a) distance traveled in the bottom region (cm), (b) total distance traveled (cm), (c) average movement speed (cm/s), (d) distance traveled in the top region (cm), (e) latency to enter the top region (s), and (f) total time in the top region (s). Zebtrack software^65^ was used to analyze the behavioral parameters of the zebrafish.

### Open Field Test in a Petri Dish

The open field test was performed in a Petri dish to evaluate the effect of aminochalcone on the locomotor activity of zebrafish. The animals were divided into groups (*n* = 6/group) that received treatment with 4AAF (0.1; 0.5; 1.0 mg/mL, 20 μL; po, respectively), diazepam (DZP; 1.0 mg/mL), or vehicle (control; 3% DMSO). One hour after treatment, the animals were individually allocated in glass Petri dishes containing the same aquarium water with the water level up to the edge of the plate, with quadrants at the bottom of the plate and dimensions of 10 × 15 cm in diameter,^50^ and the animals were filmed for 5 min for analysis. The number of line crossings between quadrants was counted using the Zebtrack software.^65^

### Spinning Task Test

The sedative effect of aminochalcone was confirmed through the Spinning Task. The test was performed according to the protocol of Blazina et al.^51^ Briefly, the groups of fish (*n* = 6) were treated with 20 μL orally of the concentrations of aminochalcone (0.1, 0.5, and 1.0 mg/mL, respectively), DMSO at 3% (vehicle, negative control), and diazepam (DZP; 1.0 mg/mL). After 60 min of administration, the animals were individually acclimated for 2 min in 250 mL beakers containing the stirring bar and 150 mL of dechlorinated water, placed on top of magnetic stirrers and isolated by black walls. After the acclimation time, the magnetic stirrer was turned on and its speed was gradually increased every 20 s until speed 3 (corresponding to 492 rpm), and the latency time for the fish to enter the whirlpool formed in the beaker after agitation at speed 3 was analyzed by blind evaluators.

### Light–Dark Test

The light/dark test was performed in aquariums measuring 30 cm × 30 cm × 15 cm, which were divided into two parts, one light and the other dark. The amount of tap water treated with a dechlorinator and without drugs added to the aquarium was equivalent to 3 cm of water in height to simulate a shallow environment different from that of the housing aquarium and induce anxiety behaviors. The fish were divided into groups of 6 animals and received 20 μL orally of the concentrations of 4AAF (0.1, 0.5, and 1.0 mg/mL, respectively). The negative and positive control groups were DMSO 3% and diazepam (DZP; 1.0 mg/mL), respectively, applied orally (20 μL). After 1 h of treatments, the analysis process occurred when the blind evaluators individually placed the fish in the light zone of the aquarium and observed the animals for 5 min for anxiolytic behavior based on the time spent in the light zone, the number of line crossings between the light and dark zone and the latency to enter the dark zone.^66^

### Mechanism of Anxiolytic Action (GABA_A_ System)

Other groups of fish (*n* = 6/group) were pretreated with flumazenil (Fmz) (0.1 mg/mL; 20 μL; i.p.), and after 15 min, one group was treated with the lowest effective concentration of 4AAF (0.5 mg/mL; 20 μL; p.o.), while the other group was administered diazepam (DZP;1.0 mg/mL, 20 μL; p.o.) positive control for sedative anxiolytic effect via GABAA.^49^ The group treated with 3% DMSO (Vehicle; 20 μL; po) was considered a negative control. After 60 min of treatment, the animals were exposed to the light/dark test described in the previous section.

### Mechanism of Anxiolytic Action (Serotonergic System)

Groups of zebrafish (*n* = 6) were pretreated with serotonergic antagonists–cyproheptadine (0.8 mg/mL; 20 μL; p.o.; 5-HTR_2A_ antagonist), pizotifen (0.8 mg/mL; 20 μL; p.o.; 5-HTR_1_ and 5-HTR_2*A*/2C_ antagonist), or granisetron (0.5 mg/mL; 20 μL; p.o.; 5-HTR_3A/3B_ antagonist). After 15 min, the lowest effective concentration of 4AAF (0.5 mg/mL; 20 μL; po) obtained in the pilot test (see the light/dark test section) was administered; 3% DMSO (vehicle; 20 μL; po) was used as a negative control. Fluoxetine (0.0125 mg/mL; i.p.) was used as a 5-HT agonist.^49^ After 60 min of treatment, animals were exposed to the light/dark test as described in the light/dark test section, and anxiolytic behavior (time spent in the light) was analyzed.

### Computational Details

The following codes were used to run the simulations: MarvinSketch version 24.1.0 (https://chemaxon.com), Avogadro version 1.2.0 (https://avogadro.cc/), AutodockTools version 1.5.6 (https://autodocksuite.scripps.edu/adt/), UCSF Chimera version 1.18 (https://www.cgl.ucsf.edu), Discovery Studio Visualizer version 21.1 (https://discover.3ds.com/discovery-studio-visualizer-download), protein–ligand Interaction Profile (https://plip-tool.biotec.tu-dresden.de/plip-web/plip/index), and pymol version 4.6 (https://pymol.org).

### Design and Optimization of Ligands

The two-dimensional structure of chalcone 4AAF was illustrated in the MarvinSketch software^67^ considering it at physiological pH, thus, the lowest energy conformer was optimized through Avogadro^68,69^ implementing the MMFF94 force field (Merck Molecular Force Field) and established for cycles of 50 interactions of the steepest descent algorithm.^70,71^

### General Molecular Docking Protocol

To evaluate the mechanism of action between 4AAF chalcone and serotonin receptors, the three-dimensional structures of 5-HTR_3A_, 5-HTR_2C_, and 5-HTR_2A_ channels were obtained through the RCSB Protein Data Bank (https://www.rcsb.org/) identified as “Cryo-EM structure of 5HT_3A_ receptor in the presence of Granisetron” (PDB ID: 6NP0),^72^ “Crystal structure of 5-HTR_2C_ in complex with ritanserin” (PDB ID: 6BQH)^73^ and “Crystal structure of 5-HTR_2A_ in complex with risperidone” (PDB ID: 6A93)^74^ in which their preparation was performed by the software AutodockTools^75^ where residues were removed and Gasteiger charges and polar hydrogen atoms were added.^76^

In the molecular docking simulations, 50 (50) independent simulations were performed, enabled by the Lamarkian Genetic Algorithm (LGA) and Exhaustiveness 64.^77^ The simulation grid was strategically positioned over the targets, fully covering the biomolecules from the axes: 159,555 (*x*), 159,367 (*y*), and 119,161 (*z*), with size parameters 126 Å (*x*), 126 Å (*y*), and 126 Å (*z*) with the 5-HTR_3A_ channel; axes: 40,574 (*x*), 33,146 (*y*), and 44,063 (*z*), size parameters 90 Å (*x*), 88 Å (*y*), and 112 Å (*z*) with the 5-HTR_2C_ channel and axes 46,155 (*x*), 1109 (*y*), and 139,552 (*z*), with size parameters 118 Å (*x*), 56 Å (*y*), and 116 Å (*z*) for 5-HTR_2A_, both for the docking simulations of the chalcone 4AAF, and for the redocking simulations of granisetron (CWB), ritanserin (E2J), and risperidone (8NU) inhibitors cocrystallized in the targets 5-HTR_2A_ and 5-HTR_3A_, 5-HTR_2C_, and 5-HTR_2A_, respectively, the technique was addressed of redocking to validate the docking simulations.

The criteria established for the molecular docking simulations were the best pose, which must establish the RMSD (Root Mean Square Deviation) statistical parameter with values up to 2 Å,^78,79^ and the affinity energy that evaluates the stability of the receptor–ligand complexes, originated with ideality parameter values lower than −6.0 kcal/mol.^80,81^ It should be noted that hydrogen bonds (H-bond) were evaluated through their intensity, providing values of the distances between the receptor and donor atoms in which Strong bonds present distances between 2.5 and 3.1 Å, average bonds between 3.1 and 3.55 Å, and weak bonds present a distance greater than 3.55 Å.^82,83^

### Hyperglycemia Induced by Sucrose (83.25 mM)

The methodology for inducing hyperglycemia in adult zebrafish was based on the study by Ranjan and Sharma^39^ with modifications. The animals (*n* = 6/group) were exposed to dechlorinated water with sucrose solution (83.25 mM/L) in their respective glass aquariums for 7 days. This dechlorinated water with a sucrose solution was replaced daily at the same time. From the eighth day onward, the animals remained in dechlorinated water without the sucrose solution and received daily treatments until the 11th day in dechlorinated water. They were subjected to oral treatments with 4AAF (0.5 mg/mL; 20 μL), DMSO 3% (vehicle; 20 μL; negative control), and metformin (5.0 mg/mL; 20 μL; positive control). After the treatments on the last day, the groups were subjected to the light–dark test (described in the previous section) to evaluate the reversal of anxiety-induced behavior by hyperglycemia. Subsequently, blood glucose levels were measured using a glucometer test strip (Active, Accu Chek) directly on the cut tail of each animal after euthanasia (described in zebrafish section).^38^ Finally, the brain and liver of the animals were collected for the analysis of reactive oxygen species (see the next section).

### Analysis of Reactive Oxygen Species (ROS)

Reactive oxygen species (ROS) analysis was based on the protocol adapted from Loetchutinat et al.^84^ Fish (*n* = 6/group) were euthanized (see zebrafish section), and then the liver and brain were collected for analysis. Afterward, the liver and brain of three zebrafish (in duplicate) were homogenized in ice-cold 40 mM Tris–HCl, pH 7.4. Eight beads were added to each tube and then macerated with a bead beater for 30 s. The homogenates were separated from the beads and centrifuged at 500 × *g* for 15 min at 4 °C, then the supernatant was collected, subjected to a new centrifugation at 12,000 × *g* for 30 min at 4 °C, and the new supernatant was collected, being kept at 4 °C. The ROS content of the tissues of animals treated with 4AAF (0.5 mg/mL), control (DMSO 3%), and metformin (5 mg/mL) was determined using DCFH-DA. Briefly, the DCFH-DA solution was incubated at 37 °C for 20 min. The fluorescence intensity of DCF was measured on a spectrophotometer (Synergy 2) with an excitation wavelength of 485 nm and an emission wavelength of 520 nm, 2 h after adding DCHF-DA to the sample. The protein concentration was determined by UV–vis light spectrophotometry at 280 nm and using a standard BSA curve.

### Seizure Crisis Induced by Pentylenetetrazol (PTZ)

Seizure induction was performed according to Siebel et al.^85^ Adult zebrafish (*n* = 6/group) were treated with 20 μL of 4AAF 0.1, 0.5, or 1.0 (mg/mL), diazepam (DZP; 1.0 mg/mL), and vehicle (DMSO 3%) p.o. After 1 h of treatment, animals were individually exposed by immersion in 10 mM PTZ readily dissolved in water in a 600 mL beaker. The beaker was then covered with a watch glass to prevent the fish from jumping. Seizure-like behavior was assessed by two pretrained observers, who scored the latency for the animals to enter each seizure stage: in stage I, the animal exhibits a dramatic increase in swimming activity; in stage II, the animal begins to exhibit whirlpool swimming behavior; and in stage III, the fish demonstrates clonus-like convulsions with loss of posture, in which the animal falls to one side and remains motionless for up to 3 s. After scoring all three test stages, the animals were sacrificed (see section on zebrafish). The animals were treated with PTZ until they reached stage III, and the latencies to the first seizure episode in stages I, II, and III were analyzed for each animal during PTZ exposure.

### Statistical Analysis

The results were expressed as the mean ± standard deviation of the mean of the in vivo tests (*n* = 6/group). The normal distribution and homogeneity of the data were analyzed using the Shapiro–Wilk test. To compare the behavioral parameters, the differences between groups were subjected to one-way ANOVA and Kruskal–Wallis, and in the antagonist experiments to two-way ANOVA, followed by Tukey’s test, using GraphPad Prism v. 8.0 software. The level of statistical significance was set at 5% (*p* < 0.05).
